# Design of agricultural wireless sensor network node optimization method based on improved data fusion algorithm

**DOI:** 10.1371/journal.pone.0308845

**Published:** 2024-11-07

**Authors:** Tang Ruipeng, Yang Jianbu, Tang Jianrui, Narendra Kumar Aridas, Mohamad Sofian Abu Talip

**Affiliations:** 1 Department of Electrical Engineering, Faculty of Engineering, University of Malaya, Kuala Lumpur, Malaysia; 2 Faculty of Languages and Linguistics, University of Malaya, Kuala Lumpur, Malaysia; 3 Department of Computer Science and Technology, School of Information Engineering, Shanghai Maritime University, Nanhui New Town, Pudong New Area, Shanghai, China; Jinan University, CHINA

## Abstract

The agricultural WSN (wireless sensor network) has the characteristics of long operation cycle and wide coverage area. In order to cover as much area as possible, farms usually deploy multiple monitoring devices in different locations of the same area. Due to different types of equipment, monitoring data will vary greatly, and too many monitoring nodes also reduce the efficiency of the network. Although there have been some studies on data fusion algorithms, they have problems such as ignoring the dynamic changes of time series, weak anti-interference ability, and poor processing of data fluctuations. So in this study, a data fusion algorithm for optimal node tracking in agricultural wireless sensor networks is designed. By introducing the dynamic bending distance in the dynamic time warping algorithm to replace the absolute distance in the fuzzy association algorithm and combine the sensor’s own reliability and association degree as the weighted fusion weight, which improved the fuzzy association algorithm. Finally, another three algorithm were tested for multi-temperature sensor data fusion. Compare with the kalman filter, arithmetic mean and fuzzy association algorithm, the average value of the improved data fusion algorithm is 29.5703, which is close to the average value of the other three algorithms, indicating that the data distribution is more even. Its extremely bad value is 8.9767, which is 10.04%, 1.14% and 9.85% smaller than the other three algorithms, indicating that it is more robust when dealing with outliers. Its variance is 2.6438, which is 2.82%, 0.65% and 0.27% smaller than the other three algorithms, indicating that it is more stable and has less data volatility. The results show that the algorithm proposed in this study has higher fusion accuracy and better robustness, which can obtain the fusion value that truly feedbacks the agricultural environment conditions. It reduces production costs by reducing redundant monitoring devices, the energy consumption and improves the data collection efficiency in wireless sensor networks.

## 1. Introduction

WSN is an important application in the agricultural environment-monitoring, which has the characteristics of low cost, self-organization and low power consumption. However, many agricultural production bases generally cover a vast area [1, 2]. In order to meet the large area monitoring demand, the monitoring devices of multiple manufacturers are purchased, but the environmental parameters monitored by different monitoring devices are often different, and the differences in individual parameters are even larger. The large amount of data with temporal and spatial similarities is generated in each network cycle, which is transmitted to sink nodes and base stations [3]. The redundant data causes congestion on the overall network channel and a sharp increase in the overall energy consumption of the network. In addition, the current agricultural WSN has problems such as limited node energy, excessive energy consumption, and small throughput. Therefore, in order to make ensure the quality of network information, It needs to study how to find suitable monitoring nodes of the monitoring area to reduces the number of environmental monitoring nodes, which can reduce the cost of monitoring devices and improve the quality of the monitoring network [4]. Data fusion technology is an information processing method that performs intelligent analysis, such as data reduction and dimensionality reduction, which can extract an information processing method that is most consistent with the real environment monitoring situation. According to predetermined rules, the multiple data is collected by sensors to comprehensively extract the estimation and decision-making specified in the monitoring task [5].

## 2. Related work

In the data fusion, some scholars have conducted research and achieved some achievements. Xia et al. [6] proposed a real-time fusion strategy for a hierarchical wireless sensor network. It optimized the quality of data and the life cycle of WSNs. The parallel computing idea can improve the computing efficiency of data fusion, which achieves high accuracy in local and global fusion centers. Sun et al. [7] proposed a multi-sensor data fusion algorithm based on trust and improved genetics. It used a data fusion algorithm based on exponential trust to fuse smooth data, and improves the crossover and mutation operations in the standard genetic algorithm to improve the convergence accuracy of the algorithm and weaken and avoid jitter problems in the optimization process. Mancipe-Castro et al. [8] used sparse models as data fusion strategies to predict environmental variables in precision agriculture. It provided a fusion strategy model and predicts the derivative trend function of humidity using the SML2010 dataset from the UCI Machine learning repository. Pasha et al. [9] proposed an Efficient Mobile Element Scheduling Protocol (EMESP), which uses a popular robot path planning algorithm (called the Bug algorithm) for data fusion to improve data transmission efficiency. Torres et al. [10] proposed a multi-level data fusion architecture (Hydra) for improving sensor accuracy and recognition. It can identify and delete outliers in sensor data, which filters them to achieve sensor data fusion and save sensor network resource consumption.

Lv et al. [11] proposed a loosely coupled extended Kalman filter algorithm for the multi-sensor fusion in agricultural scenes. It integrated the inertial measurement unit (IMU), robot odometry (ODOM), global navigation positioning system (GPS) and visual inertial odometry (VIO), which uses visualization tools to simulate and analyze robot trajectories and errors to achieve agricultural scenarios based on multi-sensor fusion. Liu et al. [12] proposed an intelligent data fusion algorithm for wireless sensor networks based on hybrid delay-aware clustering (HDC). It combined the advantages of single-layer cluster structure and multi-layer cluster structure, and adaptively selected the clustering mode of the cluster to achieve a trade-off between network delay and energy consumption through a decision function. Cao et al. [13] proposed a heterogeneous wireless sensor network data fusion algorithm based on particle swarm optimization extreme learning machine. It used the extreme learning machine method to process data collected by sensor nodes in the HWSN hierarchical routing structure. The particle swarm optimization algorithm optimized the input weight matrix and hidden layer deviation of the extreme learning machine, which improved the efficiency of wireless sensor network data fusion. Chen et al. [14] proposed a data fusion algorithm based on adaptive weighting, which calculates the variance of the data collected by each sensor and uses it as the weight factor to adjust and allocate the weight in real-time to fuse each data group.

Zhao et al. [15] proposed a weighted fusion algorithm based on abnormal data preprocessing and adaptive estimation, which has a significant effect compared with the previous random weighting method. Wang et al. [16] proposed a data fusion method based on the recursive least squares method. In order to reduce data redundancy, they applied the gray proximity theory to the self-correlation degree. They improved the correlation function so that the improved weighting algorithm of the relevance function was applied to the fusion process. Although the data fusion method proposed by the scholars as mentioned above can solve the problem of wireless sensor data fusion in some aspects, most of existing data fusion methods are proposed under the condition of ideal computing power. It does not consider the complexity of the algorithm, the actual network environment, and the energy consumption constraints of sensor nodes, so it is unsuitable for node-level data fusion of sensor networks with remote locations and limited energy resources [17, 18]. In addition, there are relatively few studies on energy fusion for agricultural sensor networks. Although Wang et al. [19] proposed the LSTM network model,but the deployment method of energy-limited sensor nodes is still constrained. Liu et al. [20] proposed a lightweight privacy-preserving trust evaluation (LPPTE) scheme. It can balance trust evaluation and privacy protection with low overhead and promote distributed data fusion in collaborative vehicle safety applications. Gao et al. [21] designed a transformer-based baseline constructed by a cross-scale mixed attention transformer (CSMFormer) and combined the information of the original scale to achieve cross-scale feature calibration for multi-source remote sensing data fusion and classification.

Therefore, in this study, it first reviews the existing data fusion algorithms, including hierarchical wireless sensor network real-time fusion strategies, multi-sensor data fusion algorithms based on trust and improve genetic algorithms, etc., and analyzes the advantages and disadvantages of each algorithm. Then it proposes a data fusion algorithm for optimal node tracking in agricultural wireless sensor networks. By introducing the dynamic bending distance in the DTW algorithm instead of the absolute distance of the correlation function, which improves the data fusion model. It can extract the fusion value of multiple same-type sensor monitoring data and find the monitoring node closest to the fusion value in the area, which reduces the number of environmental monitoring equipment and the energy loss of the network. Finally, this study designs experiments and preprocesses sensor data and compares it with other data fusion algorithms. Through the fusion results of interference-free data and interference data, the advantages of the improved algorithm in accuracy, robustness and anti-interference ability are verified, which shows that it can reduce the farm’s expenditure on environmental monitoring equipment and improves the transmission efficiency of environmental monitoring networks.

## 3. Materials and methods

### 3.1 Fuzzy association algorithm

Based on the fuzzy association algorithm, one can mine the connection between the data and then get the data fusion method. According to the degree of correlation between them, the degree of correlation is considered the basis for fusion [22]. Suppose certain data has a high degree of correlation. In that case, a larger weight should be given to the final fusion so that the influence of abnormal data with large errors on fusion estimation can be reduced. The influence of calculation value can improve the accuracy of fusion results.

Assuming that the correlation distance degree *dist*_*ij*_ between elements *p*_*x*_ and *p*_*y*_ is defined by a certain function, as shown in Eq 1:

$${dist}_{xy}=cont(p_{x},p_{y});x,y=1,2,3,\ldots .,j$$ (1)

In Eq 1, dist_xy_ represents that the correlation distance between elements *p*_*x*_ and *p*_*y*_*; cont*(*p*_*x*_,*p*_*y*_) represents that the correlation function that measures the strength of the relationship between two elements. x and y Indices of the elements. So *cont*(*p*_*x*_,*p*_*y*_) is an association function. The correlation function needs to meet the following conditions:

1. *cont*(*p*_*x*_,*p*_*y*_)*ϵ*[0,1]
2. *cont*(*p*_*x*_,*p*_*y*_) = *cont*(*p*_*y*_,*p*_*x*_)
3. *If* |*p*_*x*_−*p*_*y*_|<|*m*−*n*|*cont*(*p*_*x*_,*p*_*y*_)*>cont*(*m*,*n*), *m*, *n*, *p*_*x*_,*p*_*y*_*> 0*

When *dist*_*xy*_ = 1,*p*_*x*_ *and p*_*y*_ have the same value, *p*_*x*_ and *p*_*y*_ is strongly supported for each other; when *dist*_*xy*_≠0, the supportive degree of *p*_*x*_ *and p*_*y*_ is determined by dist_xy_; when *dist*_*xy*_ = 0,*p*_*x*_ and *p*_*y*_ have no connection with each other, *p*_*x*_ *and p*_*y*_ are completely unsupportive.

Since the signals directly collected by the environmental sensor are inherently uncertain, if the inaccurate data is directly calculated for the correlation degree, the weight distribution will be unreasonable, which eventually affects the fusion result’s accuracy. Therefore, the fuzzy association algorithm of this study uses the membership function in fuzzy theory to fuzzy the support degree of sensor data and reduce the interference of low-precision data. The logsmf function is selected as the fuzzy membership function, and Eq 2 of the membership function is as follows:

$$logsmf(x)=\frac{1}{1+e^{-kx}}$$ (2)

In Eq 2, *k* represents the parameter that adjusts the decay range of the log sigmoid function. When k ≥0, it changes the attenuation range of the logsmf function by adjusting the value of k. x represents that the input variable. Since the logsmf fuzzy membership function does not meet the second condition for constructs the correlation function mentioned above, which is *cont*(*p*_*x*_,*p*_*y*_)≠*cont*(*p*_*y*_,*p*_*x*_), the logsmf fuzzy membership function is reconstructed, and the logsmf fuzzy correlation function is established, as shown in Eq 3:

$$cont(p_{x},p_{y})=2\times {\left(1+e^{F|p_{x}-p_{y}|}\right)}^{-1}$$ (3)

In Eq 3, *F* represents the parameter that adjusts the sensitivity of the support degree. When *F ≥0*, the larger *F* is, the higher the degree of mutual support is, and the smaller *F* is, the smaller the degree of mutual support is. *p*_*x*_ and *p*_*y*_ represents the values of elements.

### 3.2 Improved fuzzy association algorithm

**Fig 1:**
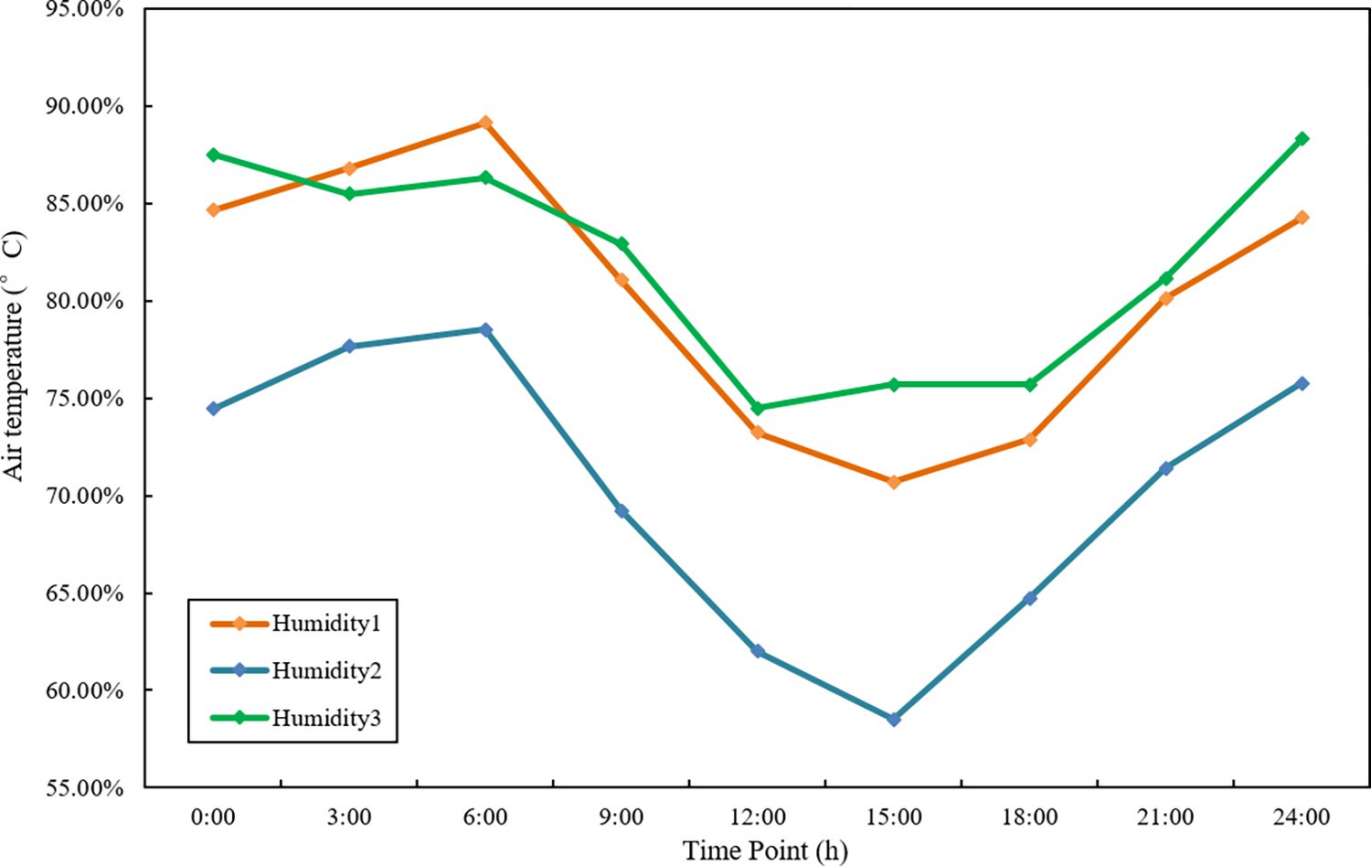
Three groups of air sensor relative humidity time series data.

The fuzzy correlation algorithm measures the absolute distance between the two sensor data at time t. If the absolute distance between two data points at this time is small, it is determined that the fuzzy correlation between the two is large; if the absolute distance between two data points is large, it is judged that the degree of fuzzy correlation between the two is small [23]. However, when this method deals with time-series sensor data, it is unreasonable to judge that the two sensors support each other only by the closeness of the data, which ignores the correlation information between the data before and after this moment. Assuming that there are three identical air humidity sensors arranged in three different positions of a greenhouse and start to monitor the relative humidity of the greenhouse, the data collection is shown in Fig1.

In Fig 1, the time series data of three air relative humidity sensor data are expressed as s1, s2, and s 3, respectively. First, judge the proximity between the sensors according to the absolute distance. After calculation, the distance between s1 and s3 at any time point is closer, and have a higher degree of consistency. During fusion, sensor 1 and sensor 3 will be given closer high weights. However, it is observed that the change trends of s1 and s2 are more similar, indicating that sensor 1 and sensor 2 have a higher consistency in the entire measurement time interval, and the data of sensor 3 may be interfered with by the external environment or even the observation target It is not the relative humidity of the air in the greenhouse, so the sensor 3 should be given a lower weight during fusion, and the fusion result will be more realistic.

Aiming at the above problems, this study introduces the DTW (Dynamic Time Warping) algorithm, which measures the difference between two sequences using dynamic programming [24, 25]. This method can map multiple points in one time series to one point in another time series to obtain the minimum distance between different series and values. Since the time axis of the sequence can be extended or shortened, the algorithm has been applied in speech recognition [26], time series similarity measurement [27, 28], and image recognition. In this study, by citing the dynamic bending distance of the DTW algorithm instead of the absolute distance in the fuzzy correlation function, the correlation degree between the sensor data in a certain period is calculated, which improves the fuzzy correlation function. It is shown in Eq 4:

$$cont(A,B)=2\times {\left(1+e^{F\times len(A,B)}\right)}^{-1}$$ (4)

In Eq 4, *len*(*A*,*B*) represents the dynamic bending distance of time series data *A = {a*_1_, *a*_2_,…,*a*_*i*_*}* and *B = {b*_1_, *b*_2_,…,*b*_*j*_*}*. The improved fuzzy correlation function has the following properties:

1. If *cont*(*A*,*B*) = *cont*(*B*,*A*), the degree of correlation between x and y is the same;
2. According to the nature of the fuzzy function, the Pick value fan of *cont*(*A*,*B*) is sho*wn as 0*≤*cont*(*A*,*B*)≤1. When *cont*(*A*,*B*) *= 0*, the correlation degree between sensors *x* and *y* is the weakest.

### 3.3 The data fusion model based on improved fuzzy association algorithm

Assuming that there are multiple sensors of the same type that simultaneously collect data on the same external factor, the data set *H*_*i*_ = {*h*_1_,*h*_2_,…,*h*_*j*_}(*i* = 1,2,….*j*) is constructed, and j is the number of sensors of the same type. The data collected by sensor x and sensor y in period T is constituted to time series (H_x_(T) and H_y_(T)). During this period, the dynamic bending distance between len(H_x_(T),H_y_(T)) is calculated. The fuzzy correlation degree between two-time data series is shown in Eq 5:

$$G_{xy}=cont\left(H_{x}(T),H_{y}(T)\right)=2\times {\left(1+e^{F\times len\left(H_{x}(T),H_{y}(T)\right)}\right)}^{-1}$$ (5)

In Eq 5, *H*_*x*_(*T*) *and H*_*y*_(*T*) represents the time series formed by data collected by sensors *x* and y during time *T*. Then the further construct the fuzzy correlation matrix is shown in Eq 6:

$$G_{xy}=\left[\begin{array}{cc} \begin{array}{cc} G_{11} & G_{12}\\ G_{21} & G_{22} \end{array} & \begin{array}{cc} \ldots & G_{1j}\\ \ldots & G_{2j} \end{array}\\ \begin{array}{cc} \ldots & \ldots \\ G_{j1} & G_{j2} \end{array} & \begin{array}{cc} \ldots & \ldots \\ \ldots & G_{jj} \end{array} \end{array}\right]$$ (6)

In Eq 6, *G*_*xy*_ represents the correlation degree between sensor *x* and all other sensors except *x* during period *T*. Therefore, the correlation degree of all sensors except sensor *x* to sensor *y* is shown in Eq 7:

$$R_{x}(T)=\sum_{y=1,y\neq x}^{j}G_{xy}/j-1$$ (7)

In Eq 7, *R*_*x*_(*T*) represents the average correlation degree of sensor x’s data with other sensors’ data during observation period *T*. It states that *0* ≤*R*_*x*_(*T*)≤*1*, where *R*_*x*_(*T*) represents the correlation value indicating the proximity of sensor *x-th* data to other sensors during observation period *T*. A higher *R*_*x*_(*T*) implies that sensor x-th data is closely related to others, while a lower value suggests significant divergence, requiring smaller weights in subsequent data fusion. However, *R*_*x*_(*T*) solely depicts the correlation between sensor *x-th* data and others during *T*. Considering potential errors in sensor manufacturing and assembly, the reliability of sensor x’s data throughout the entire T should be evaluated. When *k* is the number of data points, *P*_*x*_(*T*) = {*p*_*x*_(1),*p*_*x*_(1),…,*p*_*x*_(*k*)} represents the data collected by sensor *x* during *T*. Thus, the mean value and variance of sensor x-th data are defined as shown in Eqs 8 and 9:

$$V_{x}(T)=\frac{\sum_{t=1}^{k}p_{x}(k)}{k}$$ (8)

$$U_{x}^{2}(T)=\frac{\sum_{t=1}^{k}{\left[p_{x}(t)-V_{x}(T)\right]}^{2}}{k}$$ (9)

In Eqs 8 and 9, *V*_*x*_(*T*) represents the Mean of the data collected by sensor *x* during period *T*. $U_{x}^{2}(T)$ represents the Mean of the data collected by sensor *x* during period *T*. *p*_*x*_(*t*) represents the data points collected by sensor xx during period *T*. In sensor monitoring scenarios, environmental quantities typically exhibit smooth and continuous variations. Consequently, the collected time series data should demonstrate minimal fluctuations during the observation period. Variance serves as a measure of a sensor’s self-reliability within this period: higher variance indicates lower self-reliability, while lower variance implies greater self-reliability [29]. Following a comprehensive assessment of both the correlation between similar sensors and each sensor’s individual reliability, data fusion among similar sensors is achieved through a weighted fusion algorithm. The ultimate weighted fusion process is depicted in Eq 10:

$$\hat{P}(T)=\frac{\sum_{x=1}^{j}P_{x}(T)W_{x}(T)}{\sum_{x=1}^{j}W_{x}(T)}$$ (10)

In Eq 10, *W*_*x*_(*T*) represents the weighted value corresponding to the time series data collected by the sensor node x in the observation period T. During data fusion, sensors demonstrating high correlation and reliability should be given greater weight, so $W_{x}(T)=\frac{R_{x}(T)}{U_{x}^{2}(T)}$ is setted.

Overall, in the construction of the improved data fusion model, the first step is data collection. Multiple sensors of the same type are used to simultaneously collect data on the same external factor, creating a data set *H*_*i*_ = {*h*_1_,*h*_2_,…,*h*_*j*_}(*i* = 1,2,…,*j*). The second step is data processing. The collected data often exhibit large fluctuations and poor smoothness, affecting the accuracy of subsequent data fusion. Polynomial least squares filtering is applied to the sample data to improve stability and smoothness. The third step is to calculate DTW (Dynamic Time Warping) distance. For each sensor pair, the DTW algorithm calculates the dynamic bending distance between their respective time series data *H*_*x*_(*T*) *and H*_*y*_(*T*). This distance measures the difference between two sequences using dynamic programming, allowing for time shifts and varying speeds within the sequences. The fourth step is to compute fuzzy correlation. The fuzzy correlation degree between two-time series data is calculated using the Equation: *G*_*xy*_ = *cont*(*H*_*x*_(*T*),*H*_*y*_(*T*)).

**Fig 2:**
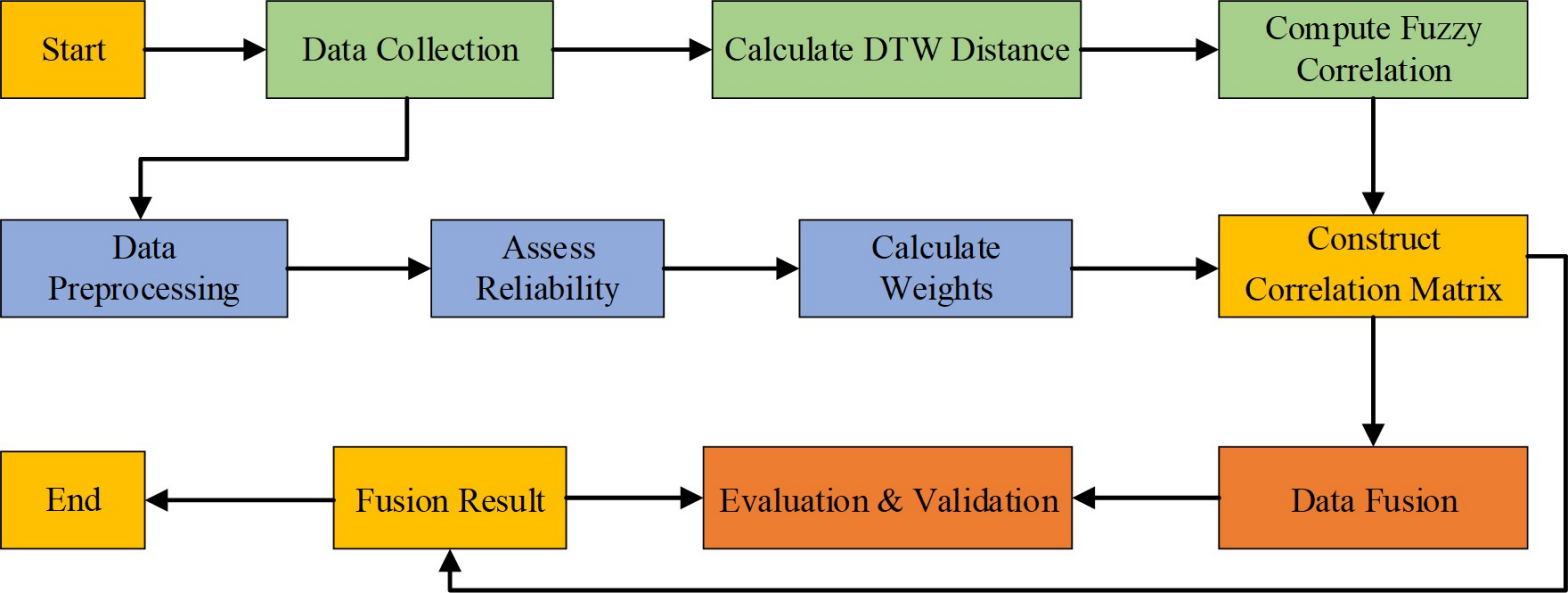
The construction process of the improved data fusion model.

The five step is to construct correlation matrix. The correlation matrix *G*_*xy*_ is constructed, representing the correlation degree between each sensor’s data and every other sensor’s data during the observation period *T*. The six step is to assess reliability. Each sensor’s data reliability is assessed by calculating the mean $V_{x}(T)=\frac{\sum_{t=1}^{k}p_{x}(k)}{k}$ and variance $U_{x}^{2}(T)=\frac{\sum_{t=1}^{k}{\left[p_{x}(t)-V_{x}(T)\right]}^{2}}{k}$ of the data collected by the sensor x during *T*. The seven step is to calculate weights. Weights are calculated based on each sensor’s correlation with others and its own reliability. The weight *W*_*x*_(*T*) for sensor x during T is given by: $W_{x}(T)=\frac{R_{x}(T)}{U_{x}^{2}(T)}$. The final step is data fusion. It involves combining the data from all sensors using the calculated weights:, which ensures that data with higher reliability and stronger correlation are given more weight in the fusion process. Fig 2 shows the construction process of the improved data fusion model.

## 4. Experimental design

In this study, the temperature data collected simultaneously by multiple air temperature sensors in continuous time is selected as the experimental data. Using MATLAB2022a software, the improved fuzzy correlation algorithm is used to fuse them, and finally the results are obtained. The experimental results mainly have the effectiveness evaluation of fusion results and the data fusion effect of faulty sensors.

### 4.1 Data collection

**Fig 3:**
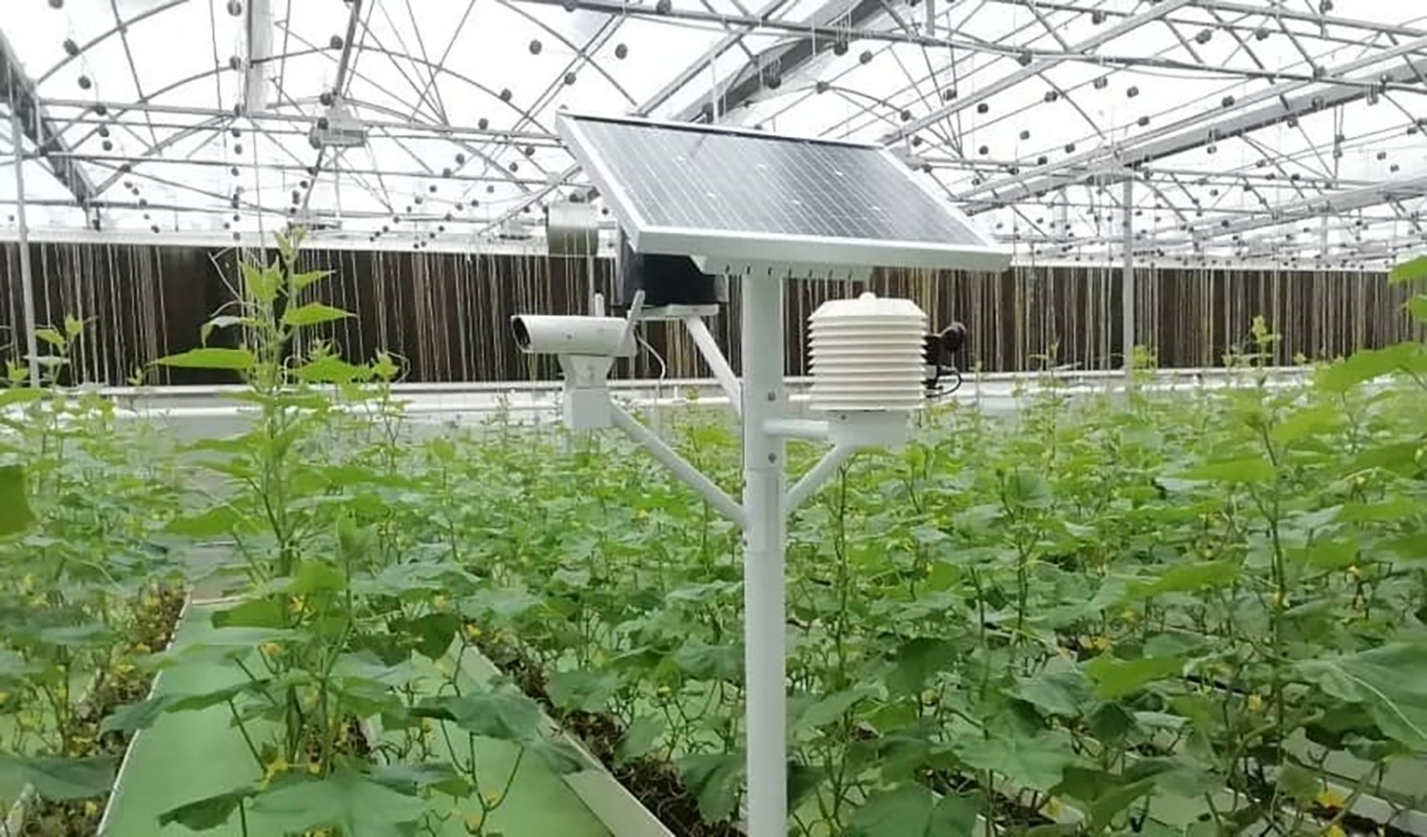
The working site of the monitoring equipment.

**Fig 4:**
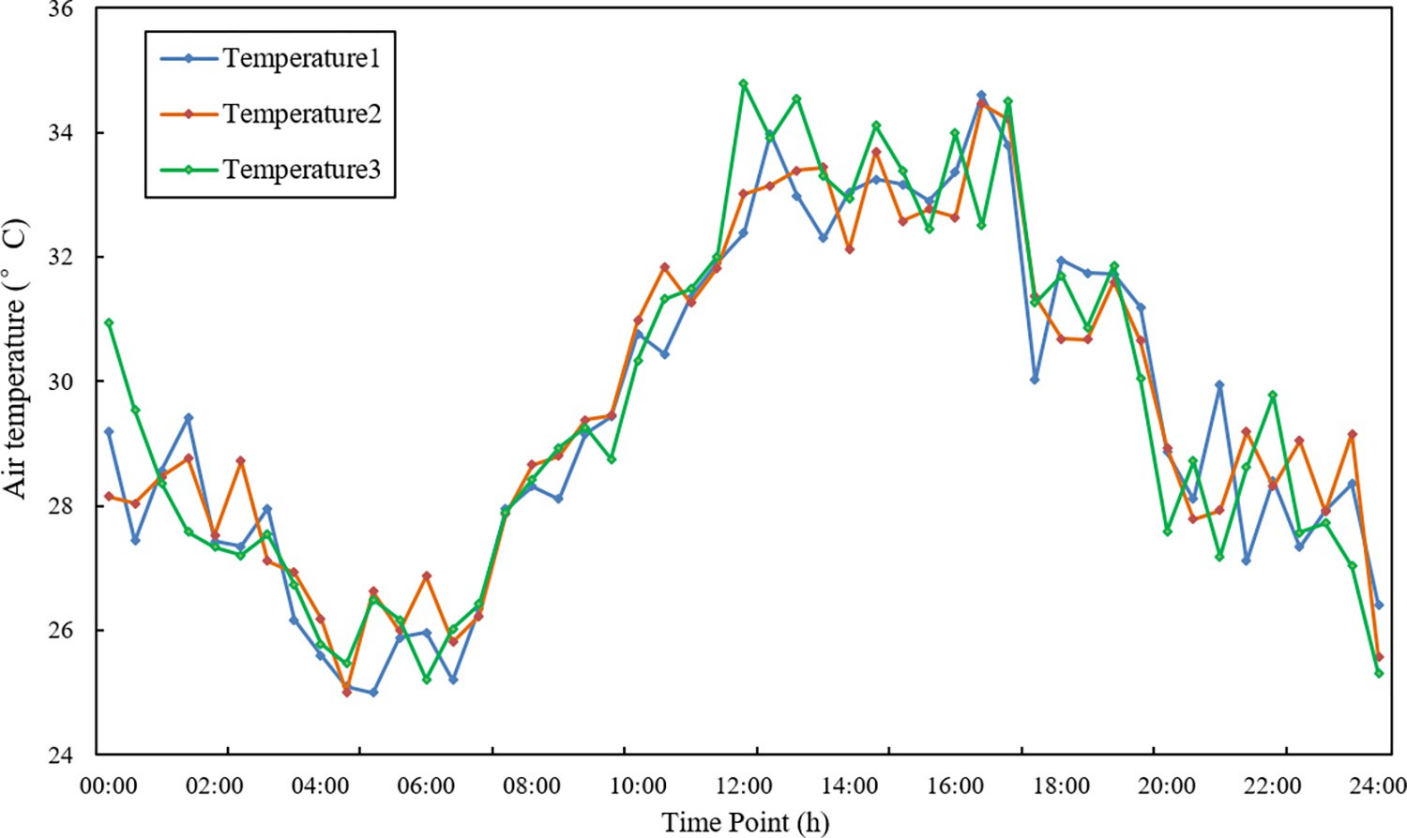
The average temperature of each period in this day.

The experimental location of this study is area A of a vegetable planting base in Qingxin District, Qingyuan City, Guangdong Province in China. The monitoring device is distributed in the middle of each plot, which is used to monitor environmental parameters such as air temperature and light (Fig 3 shows the working site of the monitoring equipment). In this study, the air sensors are taken as the research object, Table 1 shows the various experimental types and corresponding parameters. The temperature data collected by the three sensor nodes are relatively closed, and the changing trend of data sequences are more obvious and different with time. Accurate fusion of the temperature data in the three position sensors is the key to the greenhouse vegetable environment in this area,which is an important basis for making judgments about the situation. Fig 4 shows the average temperature of each period in this day.

**Table 1:** The various experimental types and corresponding parameters.

Experimental attributes	Parameters description
**Area size**	This area is divided into 3 plots, each with an area of 60 ft*60 ft
**Sampling rate**	The sampling rate of sensor nodes are set to 1
**Collection date**	Taking the data on June 15, 2023
**Collection frequency**	Each sensor collect the data every 30 minutes
**Data type**	Air temperature data
**Daily sampling volume**	Each plot generates 48 sample data

### 4.2 Data feature extraction

In this study, the data feature extraction plays a key role in ensuring accurate and efficient data fusion. The following is the data feature extraction process:

- **Data acquisition:** Multiple sensors of the same type are used to collect data on the same environmental factors, such as temperature, humidity, or soil moisture. These raw data form the basis for feature extraction.
- **Preprocessing:** Raw sensor data usually contains noise and fluctuations, which may affect the accuracy of subsequent data fusion. This study smoothes the raw data through polynomial least squares filtering to reduce noise and enhance the stability and smoothness of the data.
- **Dynamic Time Warping (DTW):** In order to accurately measure the similarity of time series data collected by different sensors, the DTW algorithm is used to map multiple points of one time series to points in another series, and the minimum distance between different series and values is calculated, taking into account time offset and speed changes.
- **Fuzzy association:** The DTW distance is then used to calculate the fuzzy association between time series data. This fuzzy association function adjusts the dynamic changes of sensor data over time and provides a more accurate measure of similarity between sensors.
- **Correlation matrix construction:** The correlation matrix represents the similarity between each pair of sensors, helping to identify the sensors with the most consistent and reliable data during the observation period.
- **Reliability Assessment:** The reliability of each sensor data is assessed by calculating the mean and variance of the data collected during the observation period. Sensors with lower variance are considered more reliable.

### 4.3 Data preprocessing

**Fig 5:**
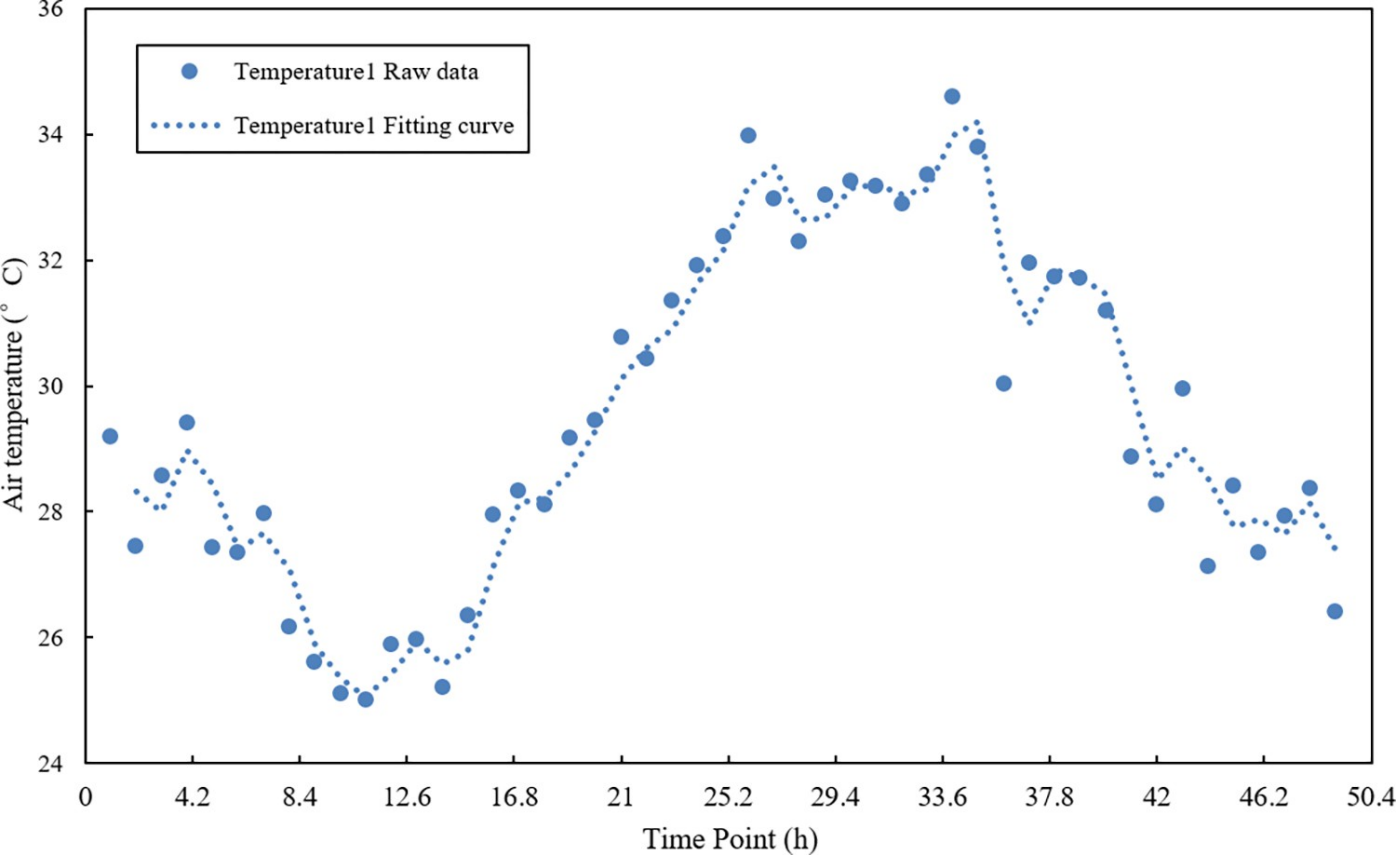
Temperature sensor 1 sample data and data curve after noise reduction.

**Fig 6:**
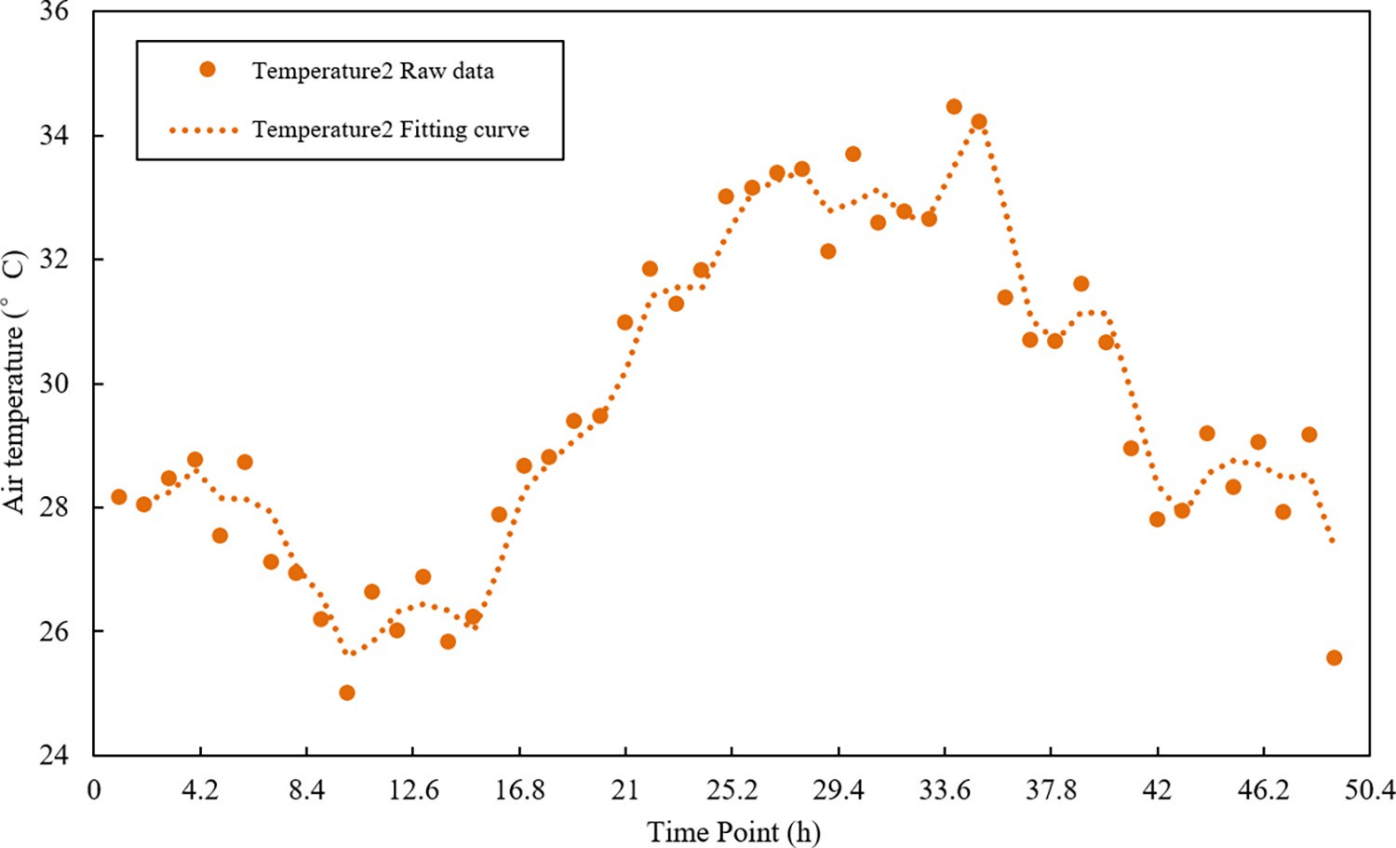
Temperature sensor 2 sample data and data curve after noise reduction.

**Fig 7:**
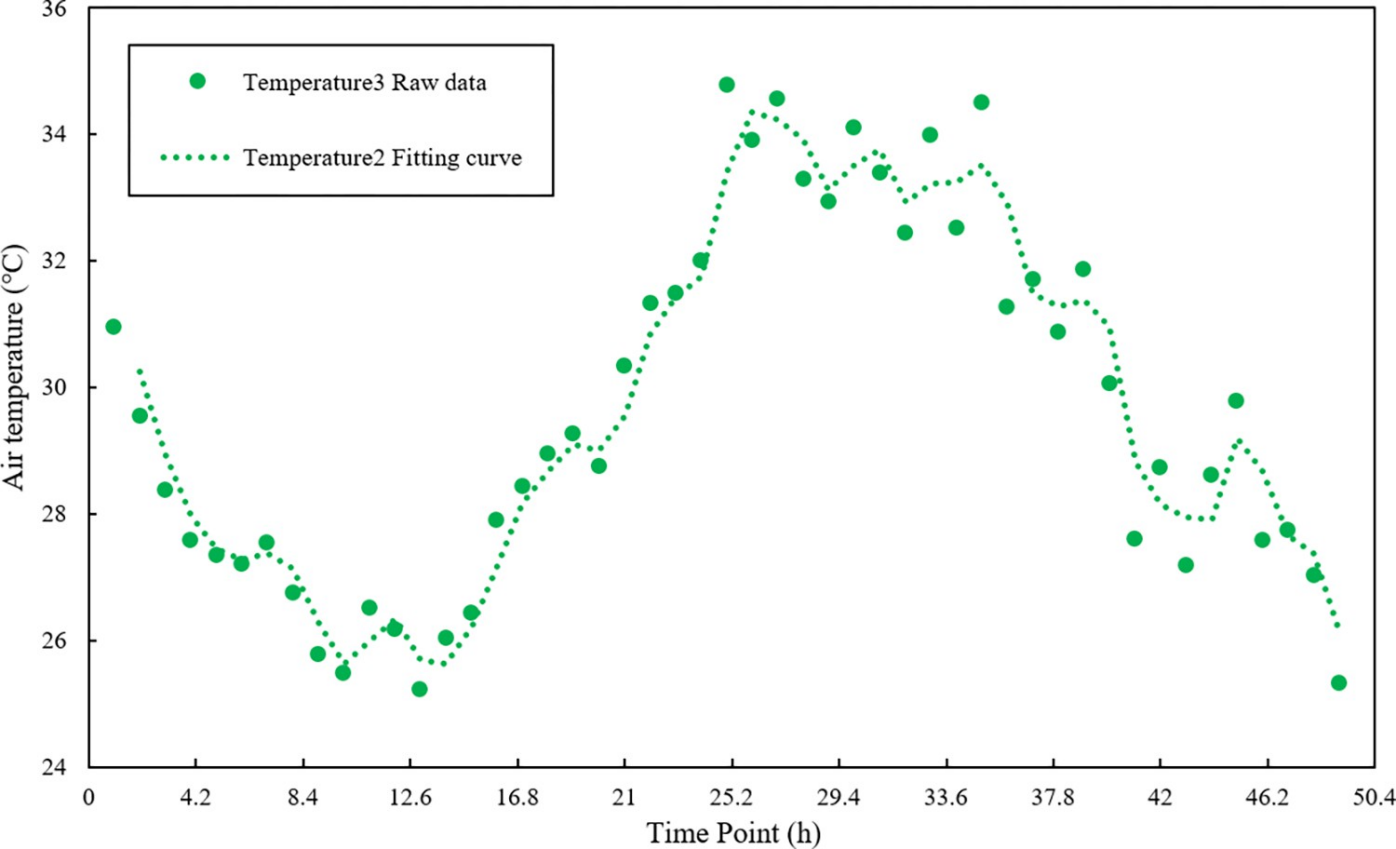
Temperature sensor 3 sample data and data curve after noise reduction.

The time series data is composed of the original sample data. It has large fluctuations and poor smoothness problems, which effects the accuracy of subsequent data fusion [30]. Polynomial least squares filtering is a method based on local polynomial least squares fitting in the time domain. This method can better preserve the local characteristics of the signal, so it is widely used in spectral analysis, smoothing and noise reduction processing of data streams [31]. Therefore, this study uses the polynomial least squares filtering method to process the sample data. After several experiments, the optimal parameters were obtained; the fitting order was 2, and the data window length was 4. The filtering effect is shown in Figs 5–7.

Figs 5–7 are the results of the temperature sample data collected by three sensor nodes using polynomial least squares filtering. After noise reduction, the temperature data has better stability and smoothness than the original sample data. The difference from the original sample temperature data is between 0–1°C, which retains the original temperature change curve. It provides the practical and reliable data to build the subsequent multi-sensor data fusion model.

### 4.4 Improved fuzzy association algorithm realization

In order to realize the improved fuzzy association algorithm, the data of the three sensor nodes is calculated in the whole period. The dynamic bending distance between two pairs of time series data can be used to replace the absolute value in the traditional fuzzy correlation function. During the monitoring period, the calculation results of the dynamic bending distance between the time series data composed of the data measured by the three temperature sensor nodes are shown in Table 2.

**Table 2:** The dynamic bending distance between three temperature time series data.

Dynamic Bending Distance	Temperature 1	Temperature 2	Temperature 3
**Temperature 1**	0	12.841	6.103
**Temperature 2**	12.841	0	6.827
**Temperature 3**	6.103	6.827	0

From Table 2, the dynamic bending distance is distributed between 6 and 13, so we should choose an appropriate F value, which makes the fuzzy correlation degree corresponding to the dynamic bending distance within a greater range of discrimination. This study selects the F value of the improved fuzzy correlation function as 0.3. Substituting the dynamic bending distance into the improved fuzzy correlation degree function, the calculation result of fuzzy correlation degree matrix is as follows:

$$G_{33}=\left[\begin{array}{ccc} 1 & 0.006 & 0.098\\ 0.006 & 1 & 0.087\\ 0.098 & 0.087 & 1 \end{array}\right]$$

In order to evaluate the performance of the algorithm in fusing sensor data, this study introduces three weights: support weight, own reliability weight and final fusion weighted value to verify the accuracy and reliability of data fusion. Support weight is used to measure the correlation of data between different sensors. It is determined by calculating the fuzzy correlation between each sensor and other sensor data. Own reliability weight measures the stability and consistency of data from a single sensor throughout the observation period, and its reliability is evaluated by calculating the variance of sensor data. Final fusion weighted value is the comprehensive weight obtained by combining the support weight and its own reliability weight. It works by multiplying these two weights to derive the weighted value of each sensor in the final data fusion process. The weight score of each node is shown in Table 3.

**Table 3:** The weighted value of each node.

temperature Weight	Temperature 1	Temperature 2	Temperature 3
**Support weight**	0.2454	0.3249	0.5071
**Own reliability weight**	0.3278	0.3346	0.3502
**Final Fusion Weighted Value**	0.2877	0.3698	0.5581

**Fig 8:**
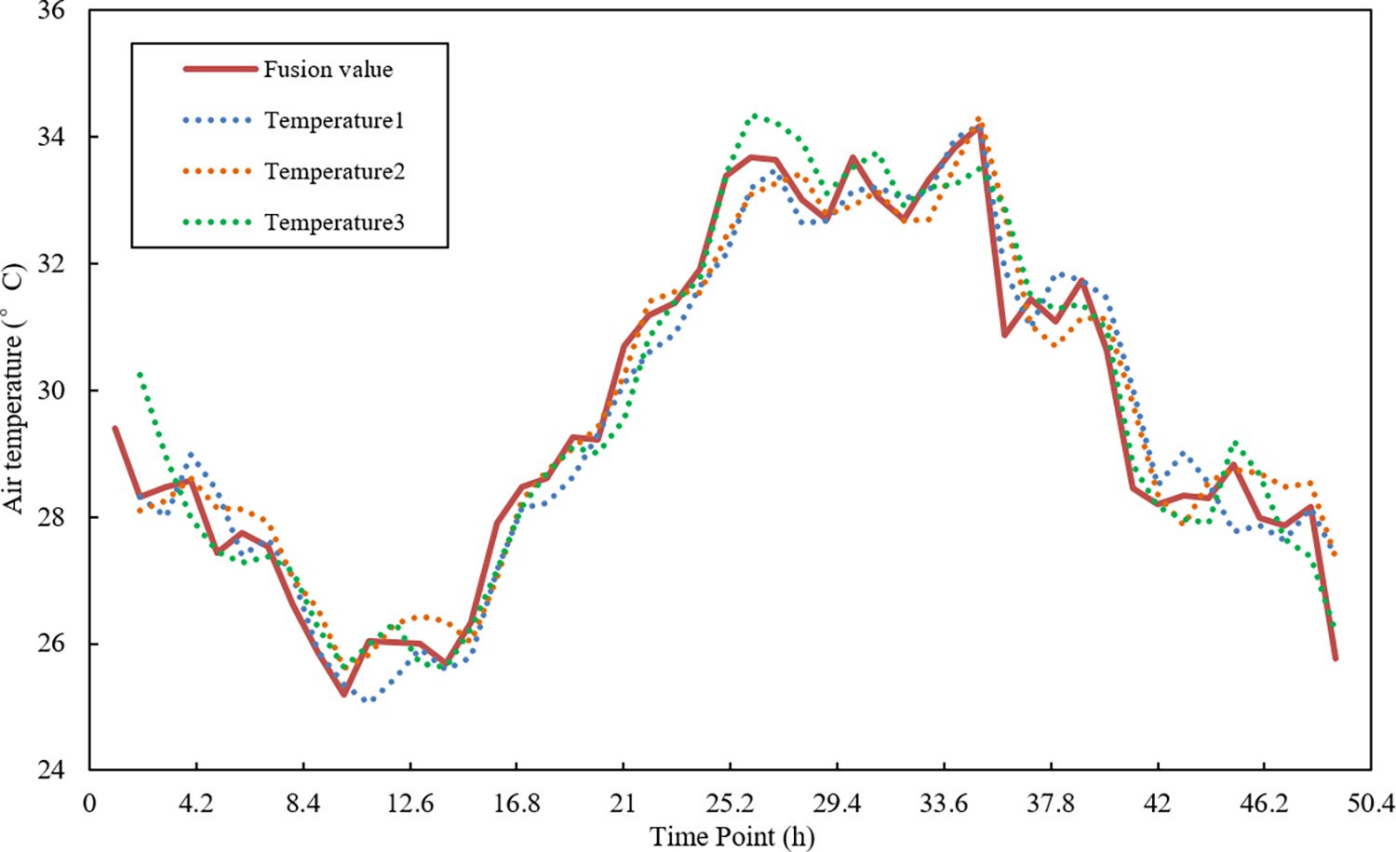
Sensor data fusion results of three nodes.

The comparison results of fusion results and original data are shown in Fig 8. After filtering and fusing the time series data of the three temperature nodes, the fusion result has eliminated the interference of abnormal data, and the fusion curve is smoother, which is the normal temperature change trend of vegetable greenhouses.

## 5. Results

In order to test the model performance of the improved fuzzy association algorithm (the algorithm proposed in this study), this study compares the improved fuzzy association algorithm with other three algorithms: the kalman filter algorithm, the unimproved fuzzy association algorithm [31], the arithmetic mean weighted algorithm [32]. Three indicators are introduced to test the performance. Among them, the variance can measure the degree of dispersion of a data set; the range indicates the gap between the maximum value and the minimum value in a set of data. These indicators measures the robustness of the fusion algorithm. If the results of each index are larger, it indicates that the robustness of the fusion algorithm is worse; otherwise, it indicates that the robustness of the fusion algorithm is better [33]. In order to fully reflect the robustness of the algorithm, this study adds the interference data in the data source and analyzes the anti-interference degree of other algorithms.

### 5.1 Fusion results without interference data

**Fig 9:**
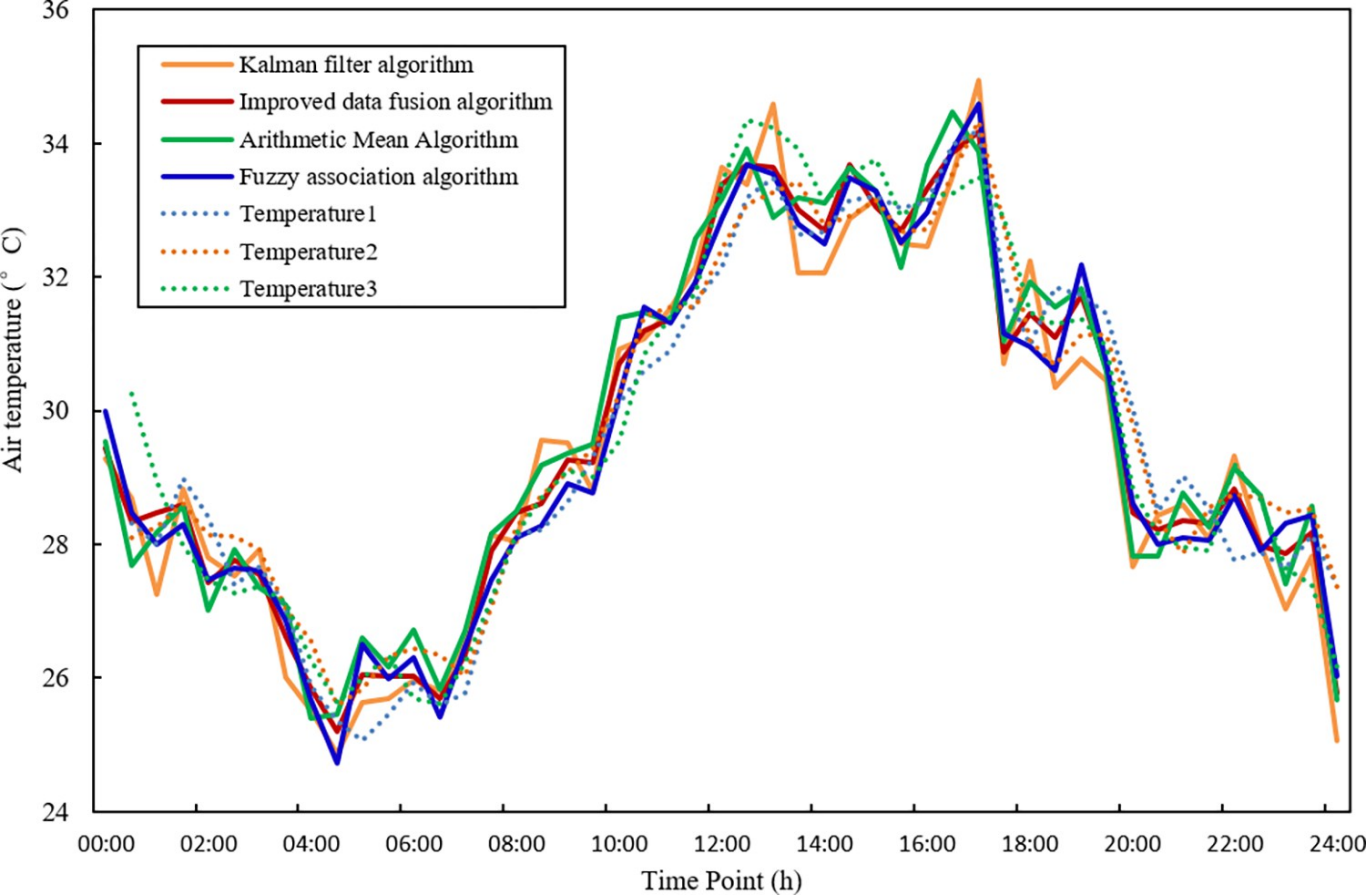
The comparison of fusion results without interference data.

They are using the improved fuzzy correlation degree to take the data collected by three temperature sensor nodes as the sample data. The fusion algorithm based on the fuzzy correlation degree, the kalman filter algorithm and the arithmetic mean fusion algorithm are used for fusion processing. The result comparison is shown in Fig 9. The evaluation indicators of the fusion results in the three methods are shown in Table 4.

**Table 4:** Comparison of fusion indicators without interference data.

Algorithms Metrics	Kalman filter algorithm	Arithmetic mean algorithm	Fuzzy association algorithm	Improved data fusion algorithm
**Average**	29.4430	29.6763	29.5072	29.5703
**Extremely bad**	9.8781	9.079	9.861	8.9767
**Variance**	2.7183	2.6609	2.6510	2.6438

In Fig 8, since the original sample data is smoother after polynomial least squares filtering, and there is no interference of abnormal data, each fusion method can make a reasonable fusion of the data in the three nodes, which is compared with the accuracy and stability of the data collected by a single temperature sensor node. Compare with the kalman filter, arithmetic mean and fuzzy association algorithm, the extremely bad of improved data fusion algorithm is 10.04%, 1.14% and 9.85% smaller than other three algorithms. The variance is 2.82%, 0.65% and 0.27% smaller than other three algorithms. The average is close to the average of the other three algorithms (the data is more uniform). It can be seen that the fusion results of the algorithm in this study are more stable and the error is smaller on the whole.

### 5.2 Fusion results with interference data

**Fig 10:**
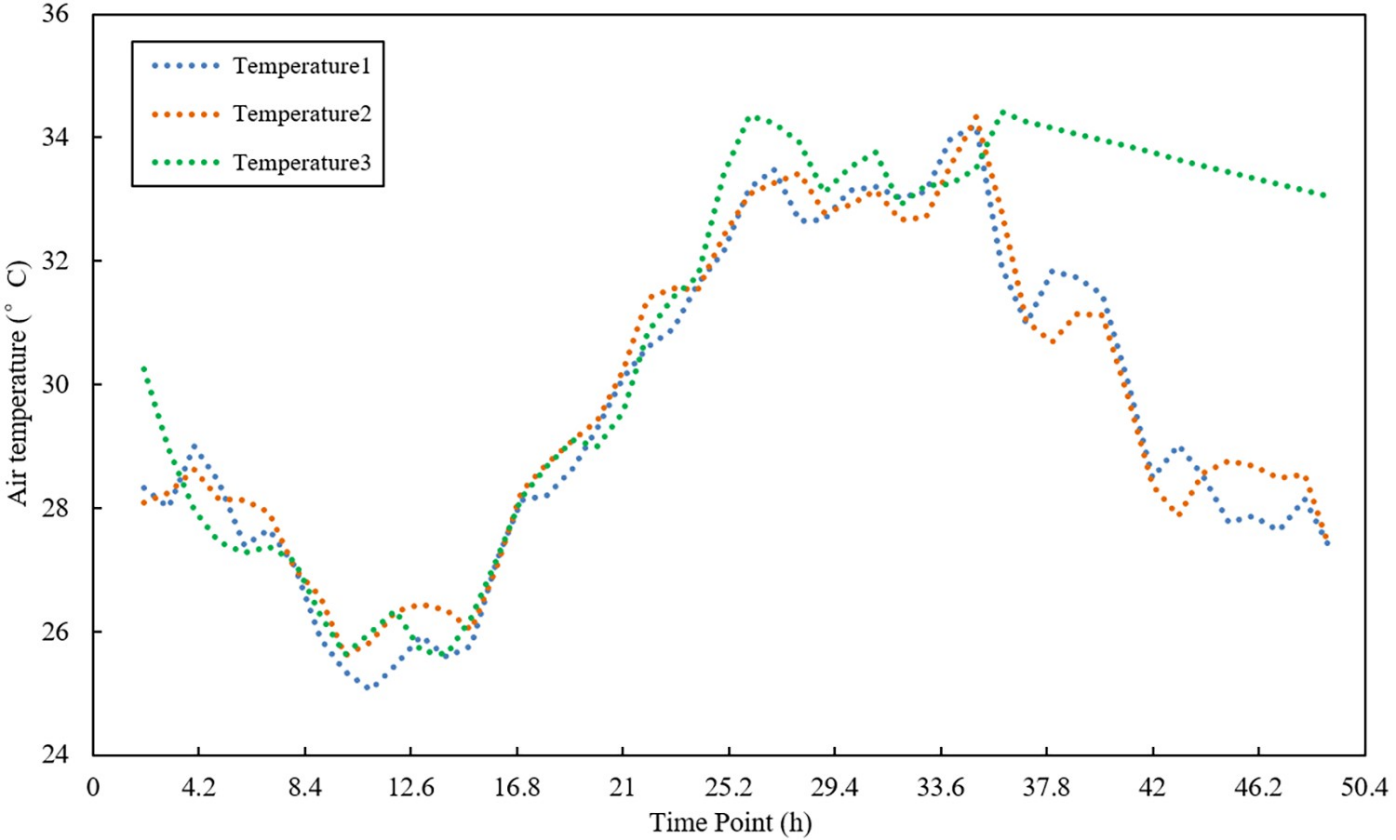
The comparison of fusion results with interference data.

During the sensor failures of actual operation often occur, such as sensor stuck, sensor constant deviation, etc., which requires the data fusion algorithm to ensure the fusion accuracy and reduce the impact of abnormal data on the fusion result. In this study, the temperature node 3 is modified to modify the sampling data at 17:30–24:00 to simulate the working state of the sensor when a constant deviation fault occurs and use it as the sample data containing interference data for simulation experiments. The abnormal data with adding interference is shown in Fig 10.

**Fig 11:**
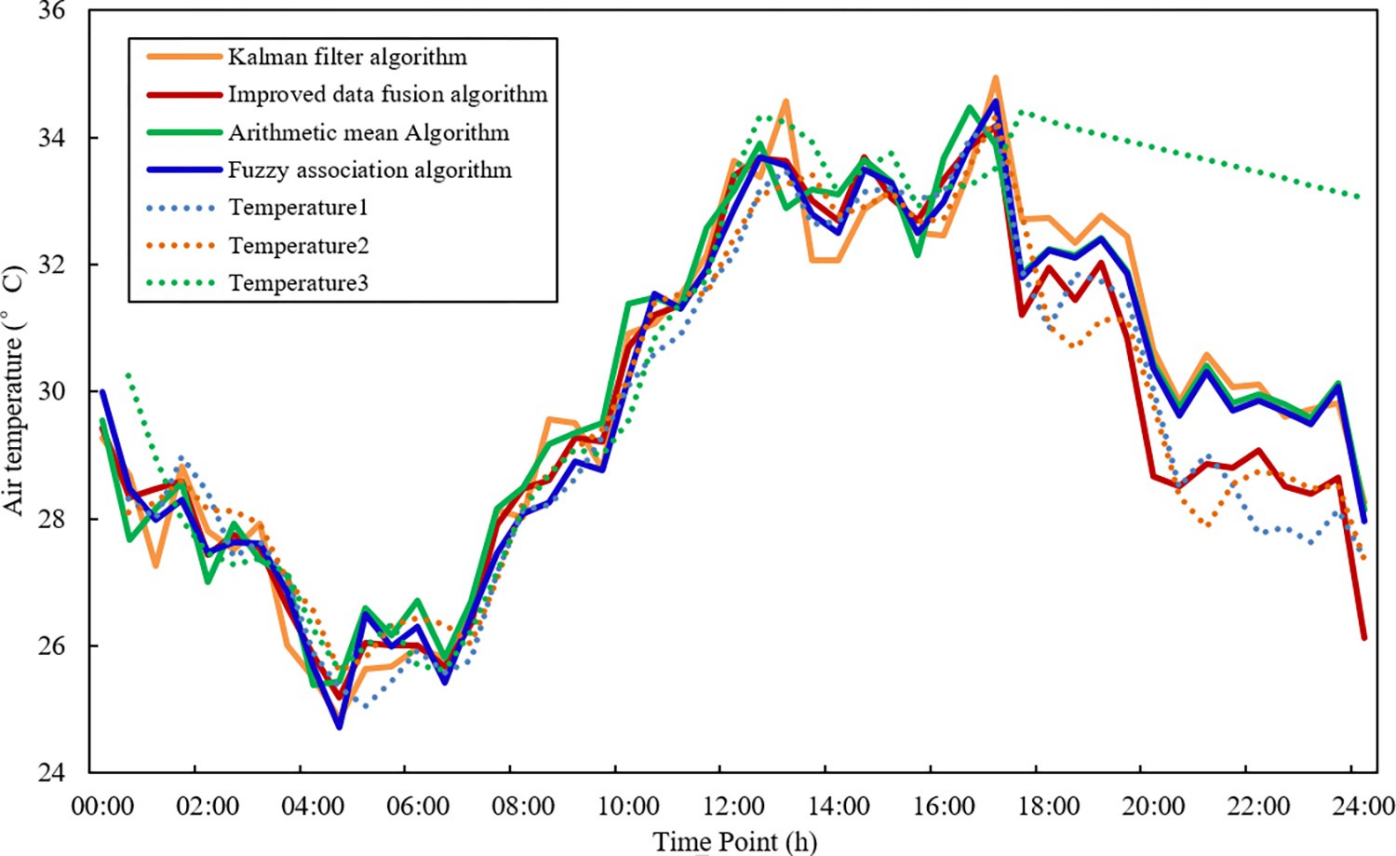
Three sensor sample data with noise data.

Three fusion algorithms are also used for data fusion, which are shown in Fig 11.

In Fig 11, since the unimproved fuzzy correlation algorithm and arithmetic mean fusion algorithm only consider the relationship between the sensor data at a single time point, so when fusion is performed within the range of abnormal data at 17:30–24:00, the fusion result curves are all biased to the sensor node curves with interference data. Compare with the kalman filter,arithmetic mean and fuzzy association algorithm,the average temperature shifted by 1.95°C, 1.39°C and 1.4°C relative to the original value without interference data, which is about 1°C higher than the 0.37°C of the improved data fusion algorithm, which shows that other three algorithms cannot well eliminate the interference of abnormal data. However, the improved data fusion algorithm considers the correlation between the data collected by each sensor during the entire measurement period, so it gives smaller weights to sensor node 3, which contains interference data. Fusion result curves are hardly affected by outlier data. It shows that the algorithm in this chapter can identify abnormal data and reduce the impact of abnormal data on the data fusion results.

## 6. Discussion

This study innovatively applies the DTW algorithm to the data fusion process of agricultural wireless sensor networks. By calculating the dynamic bending distance between time series data, it can more accurately measure the dynamic changes between sensor data and improve the accuracy of data correlation calculation. It also proposes an improved fuzzy correlation function, which combines DTW distance calculation and fuzzy logic to improve the robustness and anti-interference ability of the algorithm when processing complex environments and dynamic data by adjusting the parameters of correlation sensitivity. Finally, it uses the polynomial least squares method to smooth the data, which significantly improves the stability and smoothness of the data and reduces the impact of large fluctuations on the data fusion results; It proposes a comprehensive method based on sensor data correlation and self-reliability. The weight calculation method realizes data fusion between sensors of the same type through a weighted fusion algorithm, which ensures the reliability and accuracy of the fusion results.

In addition, the type and selection of sensors will also affect the performance of the data fusion algorithm in this study. The first is sensor quality. This study selected high-quality, low-noise and high-precision sensors to collect more reliable data and improve the overall accuracy of the data fusion process. Because poor-quality sensors will introduce noise and inaccuracy, which affects the fusion results. The second is sensor layout. The layout of sensors in the monitoring area affects their data collection, so this study uniformly covers the area to avoid environmental interference. The third is sensor calibration. This study regularly calibrates sensors to ensure the accurate measurements. Because inaccurately calibrated sensors will distort the fusion results and lead to incorrect conclusions about the monitored environment. The fourth is sensor type. Different types of sensors (such as temperature, humidity, soil moisture) have different characteristics and noise levels. After testing, the data fusion algorithm proposed in this study can adapt to processing data from various sensor types to ensure versatility in different agricultural monitoring scenarios. The last are environmental factors. The operating environment of the sensor, such as temperature changes, humidity levels and exposure to external elements, can affect the sensor performance. So the data fusion algorithm proposed in this study considers these factors to maintain good robustness.

However, current research mainly focuses on data fusion of temperature sensors, but agricultural environment monitoring is not limited to temperature, but also includes various parameters such as humidity, light, soil moisture, and carbon dioxide concentration. Future research can explore the applicability and performance of this algorithm in other types of sensor data fusion. In addition, sensors of the same type have different data characteristics and noise levels. Subsequent research will evaluate the applicability of the algorithm in different sensor types (such as soil moisture sensors, meteorological sensors, etc.) to ensure the versatility of the algorithm in multi-sensor fusion. performance and robustness. Although this study verified the performance of the algorithm under experimental conditions, subsequent implementation and testing in a real farm environment are required. Especially in large farms, the number and distribution of sensor nodes may significantly affect the performance of the algorithm. Real-world deployment and testing on large farms will provide a deeper understanding of the algorithm’s practical benefits and potential challenges. Finally, as farm sizes increase and environmental conditions diversify, algorithm scalability will become a key issue. Future research should focus on verifying the scalability of the algorithm in extremely large-scale and diverse environments, including the ability to process large amounts of sensor node data and its adaptability in highly dynamic environments.

## 7. Conclusions

This study proposes a data fusion algorithm for optimal node tracking in agricultural wireless sensor networks. It introduces the DTW algorithm into the fuzzy association algorithm, so a data fusion model based on the improved fuzzy association algorithm is constructed. By collecting temperature data from a real sensor network in a vegetable planting base and conducting simulation experiments, the data of three temperature sensor nodes are selected for data fusion. The experimental results show that the fusion result curve of the improved fuzzy association algorithm is smoother, the evaluation index is smaller than the other two algorithms, and the fusion result error is smaller. In order to verify the robustness of the improved fuzzy correlation algorithm, three data of the temperature sensor nodes is modified to simulate the constant gain fault state of the sensor in the performance of data fusion, which shows that it can better eliminate the influence of the abnormal data of the fault sensor on the fusion result, and obtains the fusion value that truly feedbacks the agricultural environment conditions. It is helpful for farm managers to find the monitoring node closest to the fusion value, which reduces redundant monitoring devices in production costs. It also reduces the network energy consumption and improves the network transmission efficiency.

## Supporting information

S1 Data (XLSX)
